# Childhood adversity and women’s cardiometabolic health in adulthood: associations with health behaviors, psychological distress, mood symptoms, and personality

**DOI:** 10.1186/s12905-019-0797-z

**Published:** 2019-07-23

**Authors:** Lotte van Dammen, Nicole R. Bush, Susanne R. de Rooij, Ben Willem J. Mol, Henk Groen, Annemieke Hoek, Tessa J. Roseboom

**Affiliations:** 1 Department of Human Development and Family Studies, Iowa State University, Ames, Iowa USA; 2 Departments of Obstetrics and Gynaecology, University of Groningen, University Medical Center Groningen, Groningen, The Netherlands; 3 Department of Epidemiology, University of Groningen, University Medical Center Groningen, Groningen, The Netherlands; 4 Departments of Psychiatry and Pediatrics, University of California San Francisco, San Francisco, California USA; 5 Division of Developmental Medicine, Center for Health and Community, San Francisco, California USA; 6 Departments of Clinical Epidemiology, Biostatistics and Bioinformatics, Amsterdam UMC at the University of Amsterdam, Amsterdam, The Netherlands; 7 Obstetrics and Gynaecology, Amsterdam UMC at the University of Amsterdam, Amsterdam, The Netherlands; 8 Department of Obstetrics and Gynaecology, Monash University, Clayton, Victoria Australia

**Keywords:** Childhood adversity, Cardiometabolic health, Health behaviors, Personality, Mental wellbeing

## Abstract

**Background:**

We tested whether childhood adversity is associated with poor cardiometabolic health in adulthood among a sample of overweight or obese Dutch women of reproductive age. Health behaviors, psychological distress, mood symptoms, or personality traits were included as potential mediators.

**Methods:**

Data came from a follow-up visit (*N* = 115), carried out in 2016/2017, of a randomized controlled lifestyle intervention trial in 577 obese infertile women. The associations between total adversity exposure score and cardiometabolic health were tested with regression models. Sleep, smoking and eating behavior, symptoms of depression, anxiety and stress, and personality traits were potential mediators.

**Results:**

Childhood adversity scores were not associated with cardiometabolic outcomes but were associated with poorer sleep quality score (M = 7.2 (SD = 3.5) for those with ≥2 types of events versus 4.8 (2.9) for those with no events; *p* = 0.022), higher external eating score (26.4 (8.7) versus 21.8 (10.3); *p* = 0.038), higher perceived stress score (17.1 (6.8) versus 12.3 (4.5); *p* = 0.016), post-traumatic stress score (1.9 (1.5) versus 0.6 (1.1); *p* < 0.001), and lower agreeableness score (28.2 (4.2) versus 30.3 (3.1); *p* = 0.035).

**Conclusion:**

Childhood adversity was associated with poorer health behaviors including sleep and eating behavior, and more stress-related symptoms, but not with women’s cardiometabolic health.

## Background

Childhood is an important developmental period during which exposure to adverse interpersonal or environmental events can meaningfully impact several domains of development and health [1, 2]. Childhood adversity is relatively common, such that in high-income countries the prevalence of having experienced at least one adverse event during childhood was estimated to be almost 40% by the WHO World Mental Health Survey [3]. More recently, in the U.S., the prevalence of exposure to violence, crime or abuse in children and youth was estimated to be as high as 58% [4]. Childhood adversity has negative effects on psychosocial and physical development [2, 5]. For example, people who experienced childhood adversity are more likely to be overweight or obese [6], have higher blood pressures [7], and an increased risk of type 2 diabetes in adulthood [8]. There are indications of increased risks of cancer and premature mortality too [2, 9–11].

Childhood adversity can come in the form of a broad array of types of events. These include witnessing a natural disaster, severe accidents, suffering from severe illness, or the death of someone close. However, being a victim of interpersonal trauma, including child abuse and sexual abuse, is more likely to result in mental health problems than other types of events [12]. It is not clear whether this association is also stronger for physical health outcomes.

Childhood adversity may directly impact cardiometabolic health. A large body of evidence suggests a direct effect of early life conditions on later development and health. The developmental origins of health and disease hypothesis [13] states that environmental stressors in early life during critical periods of development affect health and disease, such as increasing the risks of cardiovascular disease and mortality [14], through alterations in the body’s physiology, immune and vascular functioning, increased levels of stress hormones, and higher rates of glucose intolerance [1, 7, 13, 15].

Besides a possible direct effect, childhood adversity may impact cardiometabolic health in an indirect manner. For example, childhood adversity has been linked with several negative health behaviors in adulthood, such as poor sleep quality [16], smoking [17] and an unhealthy diet [6], which are known to increase the risk of cardiometabolic diseases [18–20]. This suggests the association between childhood adversity and poor cardiometabolic health may be at least partially mediated by adverse health behaviors [21].

Psychological distress and mood symptoms are other potential mediators in the association between childhood adversity and poor cardiometabolic health [22]. Childhood adversity has been associated with high levels of perceived stress later in life [23]. Depressive symptoms, anxiety symptoms [24, 25], and also early-onset psychiatric disorders like pre-school onset depression, attention-deficit disorder, oppositional defiant disorder, conduct disorder, post-traumatic stress disorder (PTSD), generalized anxiety disorder, and separation anxiety [26], each have been shown to occur more often after early life adversity, and these may increase the risk for heart disease [27–30]. Indeed, findings from a systematic review showed that psychological distress and mood symptoms partly mediate the association between childhood adversity and cardiometabolic outcomes [31].

Personality is another factor that could partially mediate the negative effects of childhood adversity on cardiometabolic health. People who have experienced childhood adversity have higher levels of neuroticism [32], and lower levels of conscientiousness and openness to experience [33]. People who have experienced childhood adversity more often have type D personality, which is a combination of social inhibition and negative affectivity [34]. Low conscientiousness and high neuroticism are linked to poorer physical health [35], and type D personality is a documented risk factor for cardiovascular morbidity and mortality [36]. Collectively, these findings point to the possibility of personality traits partially mediating the effects of early adversity on later cardiometabolic health. Indeed, there is some evidence for this. One longitudinal study demonstrated that the effect of childhood adversity on cardiometabolic health in adolescence was mediated by levels of positive personality traits, such that those who experienced greater early adversity had lower levels of positive traits [37].

Demographic characteristics may be important in childhood adversity research. For example, the prevalence of childhood adversity seems to differ among racial/ethnic groups and is related to income disparities, such that Black and Hispanic children, as well as those from low-income families, are exposed to more adversity [38]. Compared to the U.S., income disparities in the Netherlands are small and there are fewer racial/ethnic minorities, and it is important to assess the impact of childhood adversity on cardiometabolic health in countries with different racial and ethnic demographics and variations in income gaps [39]. Furthermore, the association between childhood adversity and cardiometabolic health was shown to be more pronounced in women [40], indicating that there might be sex-specific effects that merit deeper focus on female samples.

From a prevention point of view, the investigation of potential indirect effects of childhood adversity on cardiometabolic health may provide insight into potential intermediate targets for intervention, if prevention of the adversity is not possible. This could result in better cardiometabolic health outcomes in the long-term for people who have experienced childhood adversity.

In the current study, using a Dutch sample of overweight and obese women of reproductive age, we examined whether childhood adversity (as a total score, but also interpersonal victimization specifically) was associated with poorer cardiometabolic health in adulthood. We also examined whether the association between childhood adversity and later cardiometabolic health was mediated by adverse health behaviors, psychological distress, mood symptoms, or specific personality traits.

## Methods

In the original randomized controlled trial (RCT), carried out in the Netherlands, 577 obese infertile women were allocated to either a six-month lifestyle intervention or a control group. Women were eligible for participation in the RCT if they were between 18 and 39 years of age, had a body mass index (BMI) of ≥29 kg/m^2^, and were infertile. Women with severe endometriosis, premature ovarian insufficiency, endocrinopathy, untreated preexisting hypertension, or women with a history of hypertension related pregnancy complications were not eligible for participation. The RCT was carried out in 23 hospitals and resulted in 822 women who were eligible for participation, 245 of these women decided not to participate, leaving 577 women who provided written informed consent. At the time of randomization, women were approximately 30 years old, had a mean weight of 103 kg and a mean BMI of 36 kg/m^2^ (range = 29–51). Results of the primary and secondary outcomes of this trial have been published previously [41, 42] and demonstrated that rates of a vaginal birth of a healthy singleton at 37 weeks or more were not higher in the intervention group, compared to the control group. The lifestyle intervention did lead to weight loss and improved cardiometabolic health in the short-term. The study was conducted following the principles of the Declaration of Helsinki, approved by the medical ethics committee of the University Medical Centre Groningen (METc code: 2008/284) and all participants gave written informed consent.

### Questionnaires

The follow-up visit of the RCT was carried out between 3 and 8 years (mean = 5 years) after baseline assessments. The protocol of the follow-up visit has been published [43]. In short, between July 2016 and September 2017, a total of 115 women who participated in the follow-up visit filled out questionnaires regarding personality, physical health, psychological distress, mood symptoms, and life events. To evaluate adversity exposure during childhood and adolescence, the 17-item Life Events Checklist for DSM-5 (LEC-5) [44] was used. This questionnaire was slightly modified to be able to distinguish childhood adversity (between birth and 18 years of age); for events that a person experienced or witnessed, the year in which the event took place was asked and later used to calculate age at exposure. We calculated two total scores. The first score was a *total adversity exposure* score with all items summed (if a woman reported any type of event occurring once or more before the age of 19, she received a score of one for experiencing that type of event during childhood). Based on these scores, participants were then divided into three categories: a group that did not experience any type of event; a group that experienced one type of event; and a group that experienced two or more types of events. To be able to conduct sensitivity analyses to ascertain whether associations were stronger for interpersonal victimization-events, a second score, *interpersonal victimization*, was calculated. This score included physical assault, sexual assault, and unwanted or uncomfortable sexual experiences, based on previous research indicating the greater relative impact of these type of events on health [12]. This variable was scored dichotomously, such that if a woman experienced this type of event at any point during childhood she received a score of one, and if she never experienced these events she received a score of zero. Thus, a dichotomous interpersonal victimization score reflected physical and sexual assault directly experienced by the individual during childhood, and the 3-point total adversity score included those experiences as well as events that occurred more broadly in the woman’s environment during childhood.

Health behaviors were assessed across three domains. Sleep quality was measured using the Pittsburg Sleep Quality Index (PSQI), a 19-item questionnaire that has been shown to have good internal consistency (Cronbach’s alpha (α) = 0.83) [45]. Smoking behavior was assessed via one item “Are you a current smoker?” (yes or no). To assess eating behavior, the Dutch Eating Behavior Questionnaire (DEBQ) was used, which resulted in three scores: external eating, restrained eating and emotional eating [46]. External eating reflects the sensitivity to external food cues, like the presence of food or taste, restrained eating reflects dieting attitudes and behaviors, and emotional eating reflects eating as coping mechanism to handle negative emotions. The DEBQ has demonstrated high internal consistency and subscale validity [46].

Psychological distress and mood symptoms were assessed with three questionnaires. Symptoms of anxiety and depression were assessed with the 14-item Hospital Anxiety and Depression Scale (HADS), resulting in summed anxiety and depression scores, with previous reports of good reliability (α depression = 0.82; α anxiety = 0.83) [47, 48]. The primary care PTSD screen (PC-PTSD), a short 5-item questionnaire with a total summed score, was used to screen for symptoms of post-traumatic stress disorder (PTSD) [49]. This questionnaire has demonstrated excellent diagnostic accuracy [49]. Perceived stress was measured with the 10-item summed Perceived Stress Scale (PSS), a questionnaire that has demonstrated excellent reliability (α = 0.89) [50, 51].

Personality was measured with two scales. The Big Five Inventory (BFI), a 44-item questionnaire, measures five dimensions of personality: extraversion, agreeableness, conscientiousness, neuroticism and openness [52], with previous reported reliability ranging from α = 0.73 to 0.86 [53]. The Type D Scale (DS-14), a 14-item questionnaire, measures type-D personality [54] and also has demonstrated good overall reliability previously, α = 0.87 [54]. The two components of type-D personality also demonstrated good reliability in previous research; social inhibition (α = 0.86) and negative affectivity (α = 0.88).

### Physical examination to assess cardiometabolic health

Physical examinations were performed by trained research staff in a mobile research vehicle, parked near the participant’s house. Height, weight, waist- and hip-circumference were each measured twice, and a third time if there was a large difference (> 0.5 kg for weight, > 0.5 cm for height and > 1 cm for waist- and hip-circumference) between the first two measurements. After a five-minute resting period, seated blood pressure was measured three times. Fasting blood samples were drawn by trained nurses, and the biochemical analyses were performed by the AMC Clinical Chemistry Laboratory. From the fasting blood samples, continuous levels of glucose, triglycerides, high-density lipoprotein cholesterol (HDL-C) were obtained.

To assess the presence of metabolic syndrome, a reflection of composite cardiometabolic health, cut-off values for obesity, hyperglycemia, dyslipidemia (HDL-C and triglycerides) and hypertension were calculated based on the US National Cholesterol Education Program Adult Treatment Panel III (NCEP ATP III) criteria [55]. A positive classification of metabolic syndrome was based on having three or more elements either above the cut-off values, or based on pharmacological treatment for hyperglycemia, dyslipidemia or hypertension.

### Statistical analysis

**Fig. 1:**
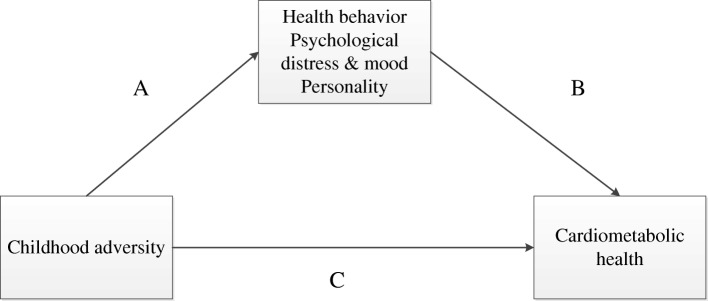
Visual model with the hypothesized main effect and mediation effects. Note: The mediators in this model include health behavior (sleep quality, smoking behavior and external, restrained, and emotional eating behavior), psychological distress and mood symptoms (symptoms of depression, anxiety, perceived stress and post-traumatic stress symptoms), and personality (openness, conscientiousness, extraversion, agreeableness, neuroticism and type D personality)

Demographic characteristics were examined with ANOVA or chi-square tests. A model with the visual representation of the associations tested is shown in Fig. 1, including the specific paths described below. ANOVA models and chi-square tests were used to test the difference in cardiometabolic health outcomes (individual measures and the composite classification score) between the groups with zero, one or ≥ 2 different types of childhood adversity, and in the sensitivity analysis between the groups with and without interpersonal victimization (path C). Second, the associations between childhood adversity levels and potential mediators (personality traits, psychological distress, mood symptoms and health behavior variables) were tested using ANOVA models and Tukey post-hoc tests (path A). The third set of analyses utilized a univariate (logistic) regression model examining the association between the mediators (personality, psychological distress, mood symptoms and health behavior) and the composite and individual cardiometabolic health outcomes (path B). To adjust for the possibility that intervention status affected the association of interest, sensitivity analyses were run that included the covariate representing randomization group in all models. All statistical analyses were performed using IBM SPSS version 24.0 (Armonk, NY, USA).

## Results

### Types of adverse events and participant characteristics

The adversity exposure groups and interpersonal victimization groups were similar in demographic characteristics (Table 1). In our sample, *n* = 69 (57.4%) reported no childhood adverse events, *n* = 29 (25.2%) reported 1 type of childhood adverse event, and *n* = 17 (14.8%) reported ≥2 types of adverse events in childhood (with *n* = 7 (6.1%) reporting ≥3 types of events). The most commonly reported adverse event was a transportation accident (*n* = 18) including car, boat, train and plane accidents, followed by physical assault (*n* = 11), sexual assault (*n* = 8), unwanted sexual experiences (*n* = 8), life threatening illness/injury (*n* = 7), severe illness or injury (*n* = 6) and sudden unexpected death of someone close (*n* = 6).

**Table 1:** Characteristics of the study participants

	Total adversity exposure				Interpersonal victimization		
	No adversity (*n* = 69)	1 type of adverse event (*n* = 29)	≥ 2 types of adverse events (*n* = 17)	*p* value	No interpersonal victimization (*n* = 93)	Interpersonal victimization (*n* = 22)	*p* value
Age (mean (SD))	36.5 (4.4)	35.0 (3.6)	34.7 (4.6)	0.168	36.1 (4.4)	35.0 (3.7)	0.316
Race (Caucasian, n (%))	63 (91)	29 (100)	17 (100)	0.121	87 (94)	22 (100)	0.221
Education level (n (%))				0.147			0.256
- Primary school	0 (0)	0 (0)	1 (5.9)		0 (0)	1 (4.5)	
- Secondary education	17 (24.6)	4 (13.8)	4 (23.5)		20 (21.5)	5 (22.7)	
- Intermediate vocational education	32 (46.4)	19 (65.5)	6 (35.3)		46 (50)	11 (50)	
- Advanced vocational education or university	16 (23.2)	6 (20.7)	5 (29.4)		22 (23.7)	5 (22.7)	

### Associations between childhood adversity and cardiometabolic health

No differences were observed in cardiometabolic health outcomes between women without adversity or women with one or two or more types of adverse childhood events (Table 2). No group differences were observed in the sensitivity analyses testing the association between interpersonal victimization and cardiometabolic health either.

**Table 2:** Childhood adversity and cardiometabolic health outcomes (path C)

	Total adversity exposure				Interpersonal victimization		
	No adversity (*n* = 69)	1 type of adverse event (*n* = 29)	≥ 2 types of adverse events (*n* = 17)	*p* value	No interpersonal victimization (*n* = 93)	Interpersonal victimization (*n* = 22)	*p* value
Anthropometrics							
BMI	35.7 (5.2)	34.7 (4.5)	36.2 (6.7)	0.593	35.7 (5.1)	34.7 (6.1)	0.437
Waist circumference (cm)	106.8 (11.2)	103.5 (10.2)	107.4 (13.5)	0.396	106.7 (11.5)	103.1 (10.3)	0.197
Blood pressure							
SBP (mmHg)	120.6 (14.4)	118.6 (13.5)	122.9 (16.2)	0.628	120.5 (14.5)	120.3 (14.2)	0.962
DBP (mmHg)	81.9 (10.1)	80.9 (8.8)	82.1 (8.8)	0.872	81.8 (9.8)	81.1 (8.5)	0.747
Composite outcome							
Metabolic syndrome	25 (41.7%)	9 (36.0%)	7 (46.7%)	0.791	34 (41.0%)	7 (41.2%)	0.987

Results are presented as mean (standard deviation (SD)) or prevalence (percentage)

*BMI* body mass index, *SBP* systolic blood pressure, *DBP* diastolic blood pressure

### Associations between childhood adversity and potential mediators

#### Total adversity score

Sleep quality scores were higher, reflecting worse sleep quality, in women with ≥2 types of childhood adverse events (7.2 (3.5)), compared to women without adversity (4.8 (2.9); *p* = 0.022). Also, higher external eating scores were observed in women with 1 type of childhood adverse event (26.4 (8.7)), compared to women without adversity (21.8 (10.3); *p* = 0.038). No differences were observed for symptoms of depression and anxiety between the groups. Levels of perceived stress were significantly higher among women with ≥2 types of childhood adverse events (17.1 (6.8)), compared to women with 1 type of childhood adverse event (12.3 (4.5); *p* = 0.016). Furthermore, higher rates of PTSD symptoms were found in women with ≥2 types of childhood adverse events (1.9 (1.5)), compared to women without adversity (0.6 (1.1); *p* < 0.001). For agreeableness, a significantly lower score was found in women with 1 type of adverse event (28.2 (4.2)), compared to the group without adverse events (30.1 (3.1); *p* = 0.035) (Table 3).

**Table 3:** Childhood adversity and health behavior, psychological distress, mood symptoms and personality (path A)

	Total adversity exposure				Interpersonal victimization		
	No adversity (*n* = 69)	1 type of adverse event (*n* = 29)	≥ 2 types of adverse events (*n* = 17)	*p* value	No interpersonal victimization (*n* = 93)	Interpersonal victimization (*n* = 22)	*p* value
Health behavior							
Sleep quality	4.8 (2.9)	5.6 (3.6)	7.2 (3.5)	0.026	5.0 (3.2)	6.8 (3.3)	0.027
Current smoker	7 (11.3%)	5 (18.5%)	4 (23.5%)	0.658	9 (10.7%)	7 (31.8%)	0.048
External eating score	21.8 (10.3)	26.4 (8.7)	26.3 (3.5)	0.038	22.6 (10.0)	28.2 (4.3)	0.011
Restrained eating score	23.4 (10.8)	24.6 (8.6)	28.1 (7.3)	0.208	24.0 (10.4)	26.0 (7.6)	0.391
Emotional eating score	26.0 (14.3)	31.0 (12.3)	30.0 (10.0)	0.183	26.8 (13.8)	32.1 (10.0)	0.096
Psychological distress and mood							
Depression symptoms	7.5 (3.5)	7.0 (2.3)	7.8 (2.8)	0.731	7.3 (3.2)	7.9 (2.7)	0.381
Anxiety symptoms	7.6 (3.1)	8.5 (3.8)	8.9 (2.9)	0.285	7.9 (3.2)	8.8 (3.5)	0.266
Perceived stress	14.0 (5.7)	12.3 (4.5)	17.1 (6.8)	0.022	13.7 (5.5)	15.5 (6.6)	0.166
PTSD symptoms	0.6 (1.1)	0.5 (1.1)	1.9 (1.5)	< 0.0001	0.7 (1.1)	1.2 (1.5)	0.063
Personality							
Type D personality	14 (20.3%)	7 (24.1%)	8 (47.1%)	0.074	20 (21.5%)	9 (40.9%)	0.059
Social inhibition	25 (36.2%)	13 (44.8%)	8 (47.1%)	0.594	36 (38.7%)	10 (45.5%)	0.561
Negative affectivity	31 (44.9%)	15 (51.7%)	12 (70.6%)	0.164	42 (45.2%)	16 (72.7%)	0.020
Openness	32.4 (5.6)	33.4 (4.0)	34.4 (3.7)	0.319	32.9 (5.3)	33.5 (3.4)	0.636
Conscientiousness	28.8 (4.1)	27.7 (4.1)	27.6 (3.8)	0.394	28.7 (3.9)	26.6 (4.3)	0.030
Extraversion	25.1 (5.4)	24.9 (4.2)	24.9 (4.1)	0.989	24.9 (5.1)	25.3 (4.0)	0.722
Agreeableness	30.1 (3.1)	28.2 (4.2)	30.4 (3.9)	0.034	30.0 (3.4)	29.1 (4.7)	0.318
Neuroticism	22.3 (5.9)	24.7 (3.7)	24.5 (4.7)	0.091	22.8 (5.4)	25.1 (4.5)	0.069

Results are presented as mean (SD) or prevalence (percentage)

PTSD post-traumatic stress disorder

#### Interpersonal adversity

The sensitivity analyses focused on exposure to interpersonal victimization during childhood paralleled the associations observed for the total adversity score described above. In addition to those associations, women with childhood interpersonal victimization were more often smokers (*n* = 7 (31.8%); *p* = 0.048) than those without interpersonal victimization (*n* = 9 (10.7%)). A positive score on the type D personality subscale negative affectivity was more prevalent in women with childhood interpersonal victimization (*n* = 16 (72.7%)), compared to those without (*n* = 42 (45.2%); *p* = 0.020). Women with interpersonal victimization reported lower conscientiousness (26.6 (4.3)), compared to women without interpersonal victimization (28.7 (3.9); *p* = 0.030).

### Associations between potential mediators and cardiometabolic health

No statistically significant associations were observed for path B between health behaviors and cardiometabolic health outcomes (shown in Table 4) or between psychological distress, mood symptoms, personality and cardiometabolic health (shown in Tables 5 and 6). Repeating the analyses with intervention randomization group as a covariate did not change the results presented in Tables 2, 3, 4, 5 and 6. Due to the lack of associations between childhood adversity and cardiometabolic health variables, no formal tests of mediation were conducted.

**Table 4:** Health behaviors and cardiometabolic health outcomes (path B)

	Sleep quality	Smoking status	External eating score	Restrained eating score	Emotional eating score
Anthropometrics					
BMI	−0.101 (0.192)	0.516 (0.701)	− 0.030 (0.061)	− 0.074 (0.057)	− 0.009 (0.044)
Waist circumference (cm)	− 0.236 (0.418)	0.962 (1.538)	− 0.092 (0.134)	− 0.189 (0.125)	− 0.064 (0.096)
Blood pressure					
SBP (mmHg)	0.465 (0.473)	−1.701 (1.851)	−0.086 (0.166)	−0.052 (0.156)	− 0.084 (0.119)
DBP (mmHg)	0.140 (0.318)	−1.179 (1.184)	−0.049 (0.112)	−0.082 (0.105)	− 0.066 (0.080)
Composite outcome					
Metabolic syndrome	OR = 1.116	OR = 0.836	OR = 1.002	OR = 0.973	OR = 1.002

Results are presented as regression coefficient(standard error(SE)) or odds ratio (OR)

*BMI* body mass index, *SBP* systolic blood pressure, *DBP* diastolic blood pressure

**Table 5:** Psychological distress, mood symptoms and cardiometabolic health (path B)

	Depression symptoms	Anxiety symptoms	Perceived stress	PTSD symptoms
Anthropometrics				
BMI	0.185 (0.187)	−0.040 (0.180)	−0.084 (0.103)	− 0.463 (0.499)
Waist circumference (cm)	0.369 (0.413)	−0.279 (0.396)	−0.293 (0.224)	− 0.712 (1.095)
Blood pressure				
SBP (mmHg)	−0.175 (0.485)	0.323 (0.463)	0.244 (0.279)	2.048 (1.281)
DBP (mmHg)	−0.095 (0.312)	0.481 (0.293)	−0.009 (0.188)	0.378 (0.867)
Composite outcome				
Metabolic syndrome	OR = 1.122	OR = 1.078	OR = 0.982	OR = 1.034

Results are presented as regression coefficient(SE) or odds ratio (OR)

*PTSD* post-traumatic stress disorder, *BMI* body mass index, *SBP* systolic blood pressure, *DBP* diastolic blood pressure

**Table 6:** Personality and cardiometabolic health (path B)

	Type D personality	Openness	Conscientiousness	Extraversion	Agreeableness	Neuroticism
Anthropometrics						
BMI	−0.668 (1.158)	−0.012 (0.124)	− 0.143 (0.150)	0.032 (0.132)	0.132 (0.175)	−0.149 (0.114)
Waist circumference (cm)	−1.478 (2.490)	0.075 (0.272)	−0.009 (0.333)	0.111 (0.290)	0.183 (0.386)	−0.392 (0.250)
Blood pressure						
SBP (mmHg)	−3.987 (3.140)	0.221 (0.319)	0.183 (0.391)	0.153 (0.341)	0.373 (0.453)	−0.123 (0.298)
DBP (mmHg)	−3.358 (2.065)	0.296 (0.202)	0.007 (0.251)	0.106 (0.218)	0.084 (0.291)	0.070 (0.191)
Composite outcome						
Metabolic syndrome	OR = 1.214	OR = 1.032	OR = 0.931	OR = 0.990	OR = 1.007	OR = 1.026

Results are presented as regression coefficient(SE) or odds ratio (OR)

*BMI* body mass index, *SBP* systolic blood pressure, *DBP* diastolic blood pressure

## Discussion

Within an understudied population of overweight and obese Dutch women of reproductive age, the present study provides evidence that childhood adversity is associated with poorer health behaviors, including sleep quality and eating behavior, and more stress-related symptoms in adulthood. However, childhood adversity was not associated with cardiometabolic health outcomes in these women.

The associations we observed between childhood adversity and various indices of health behaviors, psychological distress, and personality are in line with previous research. As in other studies, we found a higher prevalence of smoking [17], a higher prevalence of negative affectivity, one of the subscales of type D personality [34], and lower levels of conscientiousness [32, 33] among those who experienced interpersonal victimization during childhood. The associations between childhood adversity and higher levels of perceived stress and PTSD symptoms are also in line with previous research ([23, 26]), as are the associations between childhood adversity and lower sleep quality [16] and unhealthy eating behavior [6]. We found a positive association between childhood adversity and external eating behavior, where external factors, like the presence of food or the smell of food, lead to more eating [46], which is a finding not previously observed, to our knowledge. This suggests that childhood adversity may lead to more external eating behavior, which is linked to increased rates of overweight and obesity [56].

We observed associations in the analyses with childhood interpersonal victimization that were not observed in the analyses with total childhood adversity exposure. Women who had experienced interpersonal victimization were more often smokers, had more often negative affect and lower scores on conscientiousness. The observation regarding smoking behavior is in line with previous work, suggesting that interpersonal victimization affects health behavior more than other types of childhood adversity [12]. The association between childhood interpersonal victimization and personality traits (negative affect and lower conscientiousness) in adulthood has not been described previously for childhood interpersonal victimization specifically. These results suggest childhood interpersonal victimization is linked to a personality characterized by experiencing negative emotions, having less self-discipline and being less goal-oriented. Prior research suggests that these personality traits may lead to increased rates of cardiovascular disease [36, 57].

The results described in this paper regarding associations with cardiometabolic health outcomes contrast those of a large body of existing literature demonstrating the detrimental effects of childhood adversity on cardiometabolic health, cardiovascular disease and mortality [6–9]. The discrepancy between previous findings and those in the current study may be due to a number of factors. First, the types and severity of childhood adverse events reported in our sample are less severe than the types of adverse events described in the existing literature [9]. The most common adverse event reported in our study was a transportation accident, while the literature suggests that more severe events, like childhood abuse, are associated with long-term health effects [58]. However, the sensitivity analyses conducted with interpersonal victimization as a measure of those more severe events also did not reveal associations with cardiometabolic health in our sample. In addition to the apparent difference in type of events, there appeared to be a difference in the number of people exposed to several severe childhood adverse events, which was uncommon in our sample (6% had experienced ≥3 types of events). A dose-response relationship between childhood adversity and cardiometabolic health has been suggested previously, indicating that exposure to several childhood adversities is associated with poorer cardiometabolic health [9]. The small number of women with exposure to several severe childhood adverse events in our sample precluded a dose-response type of analysis. That said, it was important to discern whether the level of childhood adversity experienced by women in this understudied population played a role in the development of health behaviors, psychological distress, mood symptoms and personality to inform prevention efforts to target these risk factors for cardiometabolic disease.

Another difference between the existing literature examining the association between childhood adversity and cardiometabolic health outcomes and our study is the age of the sample. Our sample consisted of obese women who sought infertility treatment several years prior, whereas other studies conducted analyses among a general population, including predominantly people of older age [7, 9, 10]. The harmful effects of childhood adversity, partially occurring through unhealthy behaviors, psychological distress, mood symptoms and personality traits, on metabolic health and cardiovascular disease may take more time to develop. Even in at-risk populations, women are protected against cardiovascular disease before menopause, as a result of the atheroprotective effects of estrogen [59, 60]. If our study population is followed until after menopause, the effects of childhood adversity on metabolic and cardiovascular disease may be more similar to those found in previous research.

Limitations of this study should be noted. Our results may not be generalizable to a population that includes men. For example, sex-specific findings suggest that men are less vulnerable to the effects of childhood adversity on cardiovascular disease [40]. In addition, the data regarding childhood adversity were collected retrospectively in adulthood, which might have led to recall bias. Individuals experiencing stress or symptoms of depression may be more likely to report childhood adversity, which may lead to overestimating the impact of childhood adversity on the outcomes [59]. Although shorter time intervals between the event and the moment of recall are ideal, it has been suggested that reports of childhood adversity are stable over time and reliable [61]. Limitations notwithstanding, this work contributes to the literature by giving insight in the association between childhood adversity and health behaviors, psychological distress, mood symptoms and personality in an understudied population.

## Conclusion

We found that childhood adversity was associated with poorer health behaviors and greater reports of perceived stress and post-traumatic stress symptoms in adulthood. In our sample of overweight and obese women of reproductive age, no association was observed between childhood adversity and cardiometabolic health outcomes. The adverse health behaviors and increased symptoms of stress in women who experienced childhood adversity may induce poorer cardiometabolic health outcomes in the future though, warranting further follow-up of this group.

## Data Availability

A minimal dataset is available upon request.

## Abbreviations

BFI: Big five inventory
BMI: Body mass index
DEBQ: Dutch eating behavior questionnaire
DS-14: Type D scale
HADS: Hospital anxiety and depression scale
HDL-C: High-density lipoprotein cholesterol
LEC-5: Life events checklist for DSM-5
PC-PTSD: Primary care – post-traumatic stress disorder
PSQI: Pittsburg sleep quality index
PSS: Perceived stress scale
PTSD: Post-traumatic stress disorder
RCT: Randomized controlled trial
